# A Case of Resectable Single-Nodule Intrahepatic Bile Duct Adenoma

**DOI:** 10.7759/cureus.71656

**Published:** 2024-10-16

**Authors:** Hiroshi Okano, Hiroki Asakawa, Katsumi Mukai, Akira Nishimura, Takashi Hamada, Kana Asakawa, Youichirou Baba, Tetsuya Murata

**Affiliations:** 1 Gastroenterology, Suzuka General Hospital, Suzuka, JPN; 2 Gastroenterology, Suzuka general hospital, Suzuka, JPN; 3 Internal Medicine, Suzuka Kaisei Hospital, Suzuka, JPN; 4 Surgery, Suzuka General Hospital, Suzuka, JPN; 5 Pathology, Suzuka General Hospital, Suzuka, JPN

**Keywords:** bile duct adenoma, hepatic imaging, incidental findings, rare benign hepatic tumor, surgical resection

## Abstract

A 70-year-old man was incidentally diagnosed with a single hepatic mass lesion in his right hepatic lobe during a computed tomography scan. The lesion exhibited consistent enhancement with contrast agents on computed tomography (CT), magnetic resonance imaging (MRI), and hepatic arterial angiography. While a definitive diagnosis could not be made preoperatively, the lesion was surgically resected due to its slight enlargement over two months, suggesting a potential malignancy. Pathological examination revealed the lesion to be a bile duct adenoma (BDA). The BDA was characterized by dense proliferative small gland cavities containing several to dozens of cells. Immunohistochemical staining showed positive CK7 and negative p53. The patient remains alive and free of recurrence five years after hepatectomy. Although BDAs are rare benign hepatic tumors, they carry a risk of harboring or developing malignant tissue, such as cholangiocarcinoma. Therefore, BDAs or lesions suspicious of BDA should be surgically resected or closely monitored.

## Introduction

An intrahepatic bile duct adenoma (BDA) is a rare benign hepatic tumor that is incidentally discovered during intra-abdominal surgery, autopsy, or imaging examination due to other organs’ disease followed-up [1-8]. The majority of BDA cases occur in Caucasians and Afro-American people, while cases in Asians are less common [1]. The prognosis for BDA is favorable, as 30 of 38 cases remained alive after resection and were followed up for an average of 54.6 months. None of the eight deceased cases showed evidence of BDA recurrence in autopsy examinations [1]. Another report showed that 3/4 of cases were recurrence-free, while the remaining case was complicated by the recurrence of hepatocellular carcinoma (HCC), not BDA [4]. Meanwhile, the co-existence of BDA and cholangiocarcinoma or the possibility of a BDA-cholangiocarcinoma sequence is reported [3,9]. Most BDAs have a diameter ranging from 1 to 20 mm [1], with their origin being the cholangiocytes [10]. However, in rare cases, exceptionally large BDAs with a diameter as great as 92 mm have been documented [11]. Moreover, most cases of BDA have undergone surgical resection due to the difficulty of differential diagnosis from other malignant hepatic tumors, for example, hepatocellular carcinomas, cholangiocarcinoma, or metastatic tumors, even if BDA itself is no malignancy. The absence of specific imaging findings or diagnostic markers poses significant challenges in differentiating BDAs from other malignant liver tumors. In addition, some BDAs are known to exhibit neoplastic potential, followed by classical peripheral intrahepatic cholangiocarcinoma, and BDAs are associated with histologic features of malignancy or borderline lesions [10]. We experienced one resectable single nodule intrahepatic BDA case and made various modalities’ examinations, including ultrasonography, computed tomography, magnetic resonance imaging, and abdominal angiography for the BDA before the surgical resection. Herein, we report this case of BDA as a rare benign hepatic mass, comparing imaging data and pathological findings.

## Case presentation

A 70-year-old man was referred to the gastroenterology department of our hospital due to a hepatic mass detection by his chest computed tomography, which showed its lesion at the hepatic S8 segment incidentally. He had no symptoms, and his laboratory data showed mild hepatic enzyme elevations, hyperlipidemia, and diabetes mellitus (Table 1).

**Table 1:** Laboratory data in reference to the gastroenterology department CBC: complete blood count; WBC: white blood cells; RBC: red blood cells; PT: prothrombin time; TP: total protein; Alb: albumin; AST: aspartate aminotransferase; ALT: alanine aminotransferase; LDH: lactate dehydrogenase; ALP: alkaline phosphatase; g-GT: g-glutamyltransferase; T-Bil: total bilirubin; ChE: cholinesterase; T-Chol: total cholesterol; BUN: blood urea nitrogen; Crea: creatinine; eGFR: estimated glomerular filtration rate; FBS: fasting blood sugar; HbA1c: hemoglobin A1c; CEA: carcinoembryonic antigen; CA19-9: carbohydrate antigen 19-9; AFP: alpha-fetoprotein; PIVKAII: protein induced by vitamin K absence or antagonist-II; HBsAg: hepatitis B surface antigen; HBsAb: hepatitis b surface antibody; IgG-HBc: Immunoglobulin G antibody to the hepatitis B core antigen; HCVAb: anti-hepatitis C virus antibody; HIV: human immunodeficiency virus; IgG: Immunoglobulin G; IgA: Immunoglobulin A; IgM: Immunoglobulin M. ^#1^serum ALP levels measured using the International Federation of Clinical Chemistry and Laboratory Medicine (IFCC) method could be calculated as 0.34 times the ALP levels measured using the Japan Society of Clinical Chemistry (JSCC) method.

	Data	Reference
CBC		
WBC (/mL)	9300	3900–9800
RBC (/mL)	470 × 10^4^	427–570 × 10^4^
Hemoglobin (g/dL)	15.9	13.5–17.6
Hematocrit (%)	44.0	39.8–51.8
Platelets (/mL)	103 × 10^4^	130–369 × 10^4^
Coagulation		
PT (%)	100	70–130
Chemistry		
TP (g/dL)	8.0	6.5–8.5
Alb (g/dL)	4.6	4.1–5.3
AST (IU/L)	47	10–35
ALT (IU/L)	68	10–35
LDH (IU/L) (IFCC)	201	124–222
ALP (IU/L) (IFCC)	77^#1^	72–113
g-GT (IU/L)	109	8–60
T-Bil (mg/dL)	1.1	0.2–1.3
ChE (IU/L)	465	229–520
T-Chol (mg/dL)	234	150–219
BUN (mg/dL)	10.5	9.6–22.0
Crea (mg/dL)	0.69	0.50–1.10
eGFR (mL/min/1.73 m^2^)	86.01	0.00–0.30
FBS (mg/dL)	138	70–109
HbA1c (%)	6.8	4.6–6.2
Tumor markers		
CEA (ng/mL)	3.3	0–5
CA19-9 (U/mL)	68	0–37
AFP (ng/mL)	4.8	0.0–10.0
PIVKAII (mAU/mL)	25	0–39
Viral markers		
HBsAg	0.10	0.00–0.99
HBsAb	51	0–10
IgG-HBc	(+)	
HCVAb	0.1	0.0–0.9
HIV	(-)	
Serology		
IgG (mg/dL)	1399	870–1700
IgA (mg/dL)	397	110–410
IgM (mg/dL)	87	35–220

**Figure 1:**
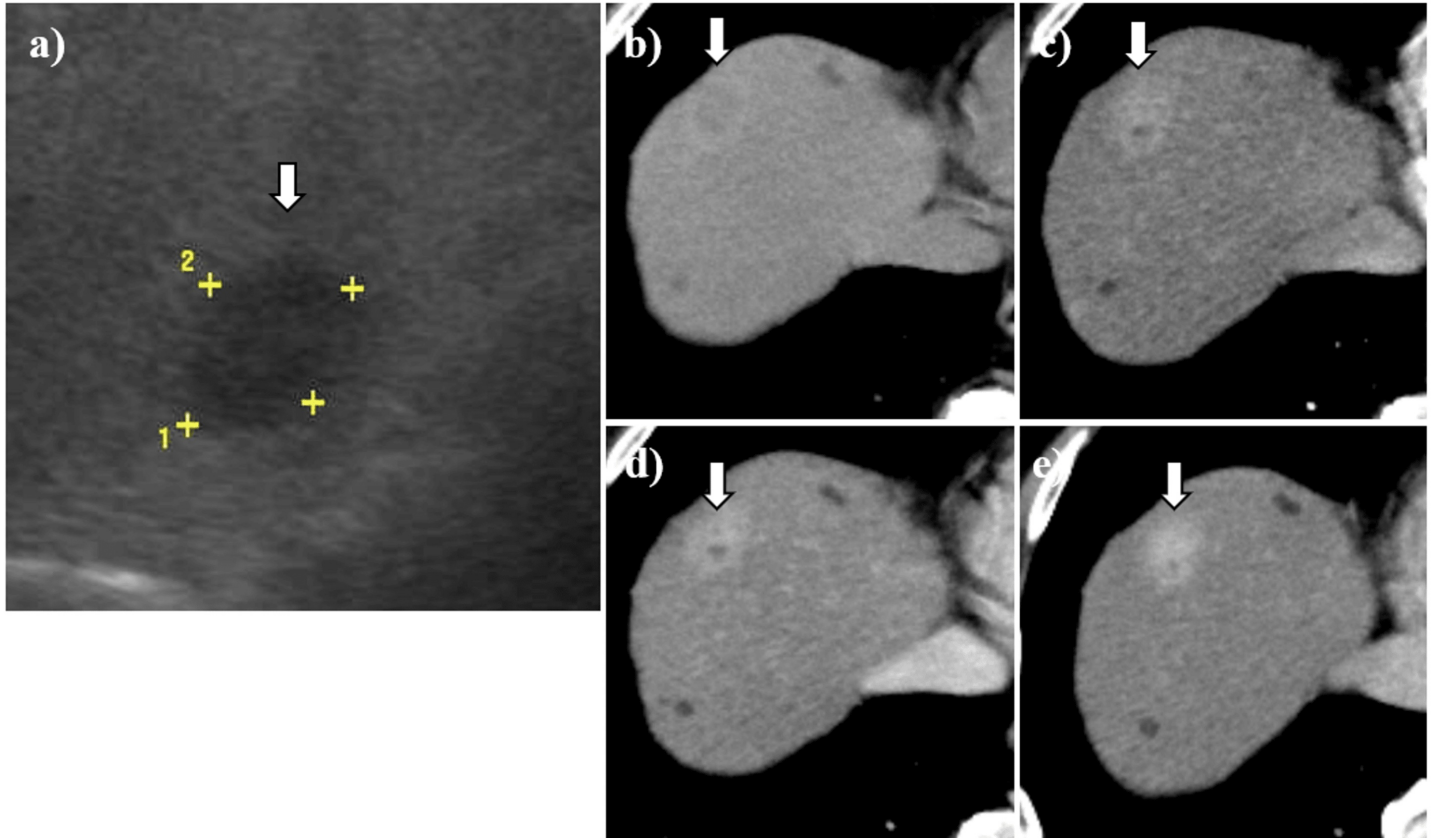
The imaging of abdominal ultrasound sonography and computed tomography. (a) Abdominal ultrasound sonography showed the hypoechoic area (arrows) (21 mm × 15 mm in diameter) as a background with hyper-echogenicity due to fatty liver change. (b) Abdominal plain CT showed a lower density nodule with slightly higher density hazy border (arrows). (c) Contrast-enhanced abdominal CT in the arterial phase showed hyperenhancement effect for the hepatic nodule with a part of inner non-enhancement area (arrows). (d) and (e) The hyperenhancement effect of the hepatic nodule continued in portal and equilibrium phase (arrows).

**Figure 2:**
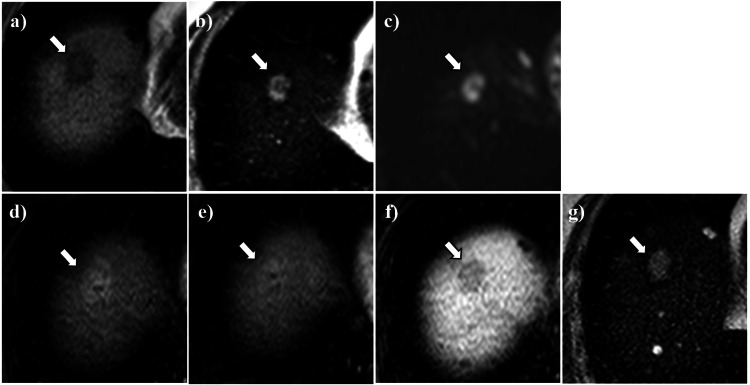
Abdominal magnetic resonance imaging. Abdominal magnetic resonance imaging (MRI) of the hepatic nodule (arrow). The hepatic nodule appeared hypointense on T1-weighted (a) image and hyperintense with hypointense area of its center on T2 (b) and diffusion-weighted (c) image. On gadolinium-ethoxybenzyl-diethylene-triamine-pentaacetic acid (Gd-EOB-DTPA)-enhanced arterial phase (d) the lesion showed hyperenhancement with hypointense area of its center that persists through equilibrium (e) phases. On Gd-EOB-DTPA-enhanced in the hepatobiliary phase showed hypointense (f). The follow-up MRI on the T2-weighted image after two months showed the hyperintense hepatic nodule slightly enlarged (g).

On abdominal ultrasound sonography, the hepatic lesion was described as a 21 mm × 15 mm hypoechoic mass in diameter in the right lobe, surrounded by a diffusely hyperechogenic background reflecting fatty change (Figure 1a). While the plain computed tomography (CT) showed a lower-density nodule with a slightly higher-density hazy border in the right hepatic lobe (Figure 1b), the contrast-enhanced CT showed enhancement with a part of the inner non-enhancement area in all phases, including the arterial phase, portal phase, and equilibrium phase (Figure 1c-1e). In the magnetic resonance imaging (MRI), the hepatic lesion was described as a hypointense mass lesion on the T1-weighted image (Figure 2a) and a hyperintense mass lesion with a hypointense area of its center on T2 (Figure 2b) and diffusion-weighted image (Figure 2c). On gadolinium-ethoxybenzyl-diethylene-triamine-pentaacetic acid (Gd-EOB-DTPA)-enhanced imaging, likely to that of contrast-enhanced CT imaging, the hepatic lesion showed hyperintensity with a hypointense area of its center in the arterial phase (Figure 2d), and its effect persisted in the equilibrium phase (Figure 2e). However, the hepatic lesion did not show the intake of Gd-EOB-DTPA in the hepatobiliary phase (Figure 2f).

**Figure 3:**
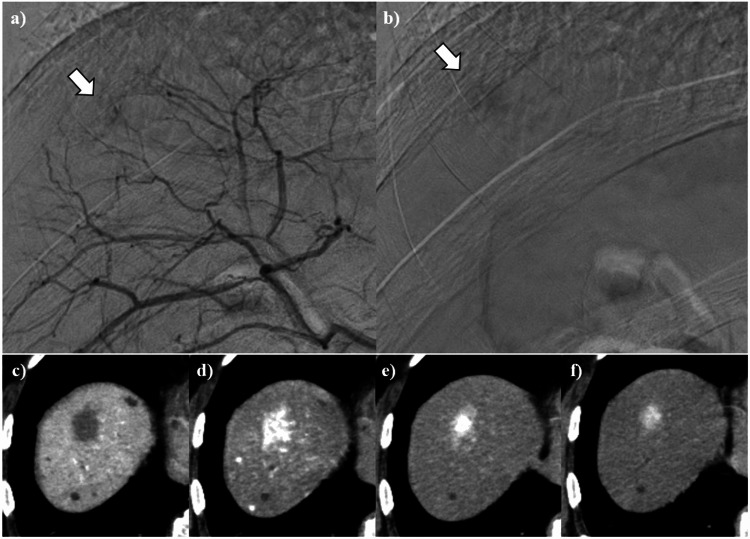
Hepatic arterial angiography. Hepatic arterial angiography of the hepatic nodule. The lesion showed persistent enhancement from the early to late phase (arrows) (a,b). CT angiography showed the lesion non-enhancement in the portal phase (c), while the lesion showed persistent enhancement during all hepatic arterial phases: (d) the early, (e) the middle, and (f) the delayed components.

As no 18F-fluorodeoxyglucose (FDG) uptake was observed in the hepatic lesion on positron emission tomography (PET)-CT (data not shown), we decided to follow up on the hepatic lesion and performed an MRI two months later. The follow-up MRI on the T2-weighted image showed the slightly enlarged hyperintense hepatic nodule and the diminished hypointense area of its center (Figure 2g). The patient remained asymptomatic at this time point, similar to the initial diagnosis. The abdominal angiography was operated on to investigate the hepatic lesion further due to the emergence of the malignant possibility from the lesion’s enlargement and its signal intensity exchange within the mass over two months. The hepatic arterial angiography showed persistent enhancement of the hepatic lesion from the early to late phase (Figure 3a-3b). CT angiography showed the lesion non-enhancement in the portal phase (Figure 3c) and persistent enhancement during all the hepatic arterial phases (Figure 3d-3f).

The hepatic lesion was suspected to be a malignant neoplasm due to the findings of imaging studies, including a slight increase in volume over two months. Based on the imaging findings, the differential diagnosis included atypical hepatocellular carcinoma, cholangiocarcinoma, combined hepatocellular and cholangiocarcinomas, epithelioid hemangioendothelioma, and other entities. While the patient refused a biopsy targeted at the hepatic lesion, he consented to a partial hepatectomy for the diagnosis and treatment of the hepatic lesions.

**Figure 4:**
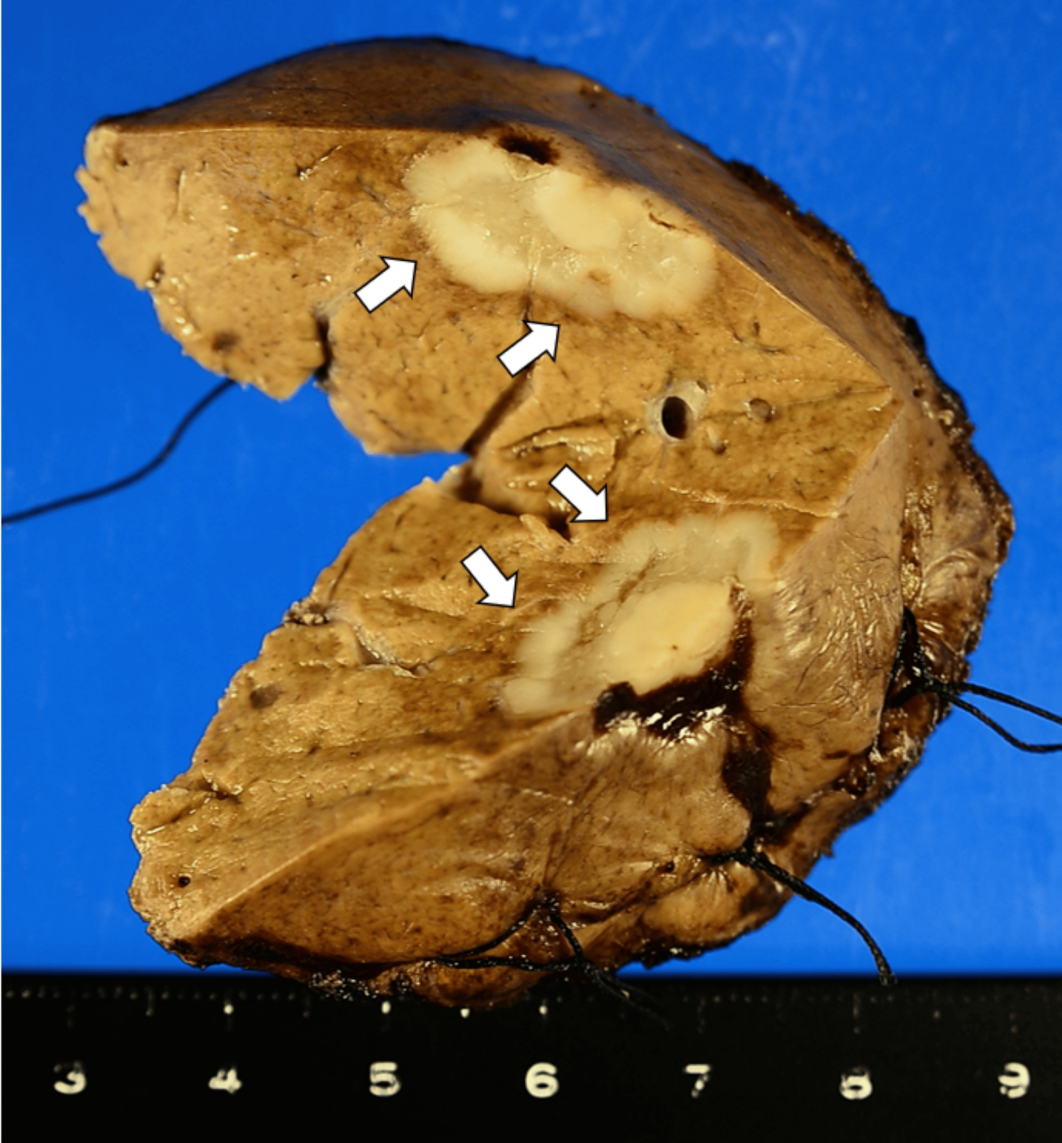
Gross specimen of the resected tumor. Gross specimen of the liver tumor obtained at surgery. The arrows indicate the hepatic tumor. The 1.7 cm × 1.5 cm × 1.1 cm tumor was well-demarcated and located beneath the hepatic capsule. The tumor-cut surface was white and solid, with some areas exhibiting a pale yellow hue.

**Figure 5:**
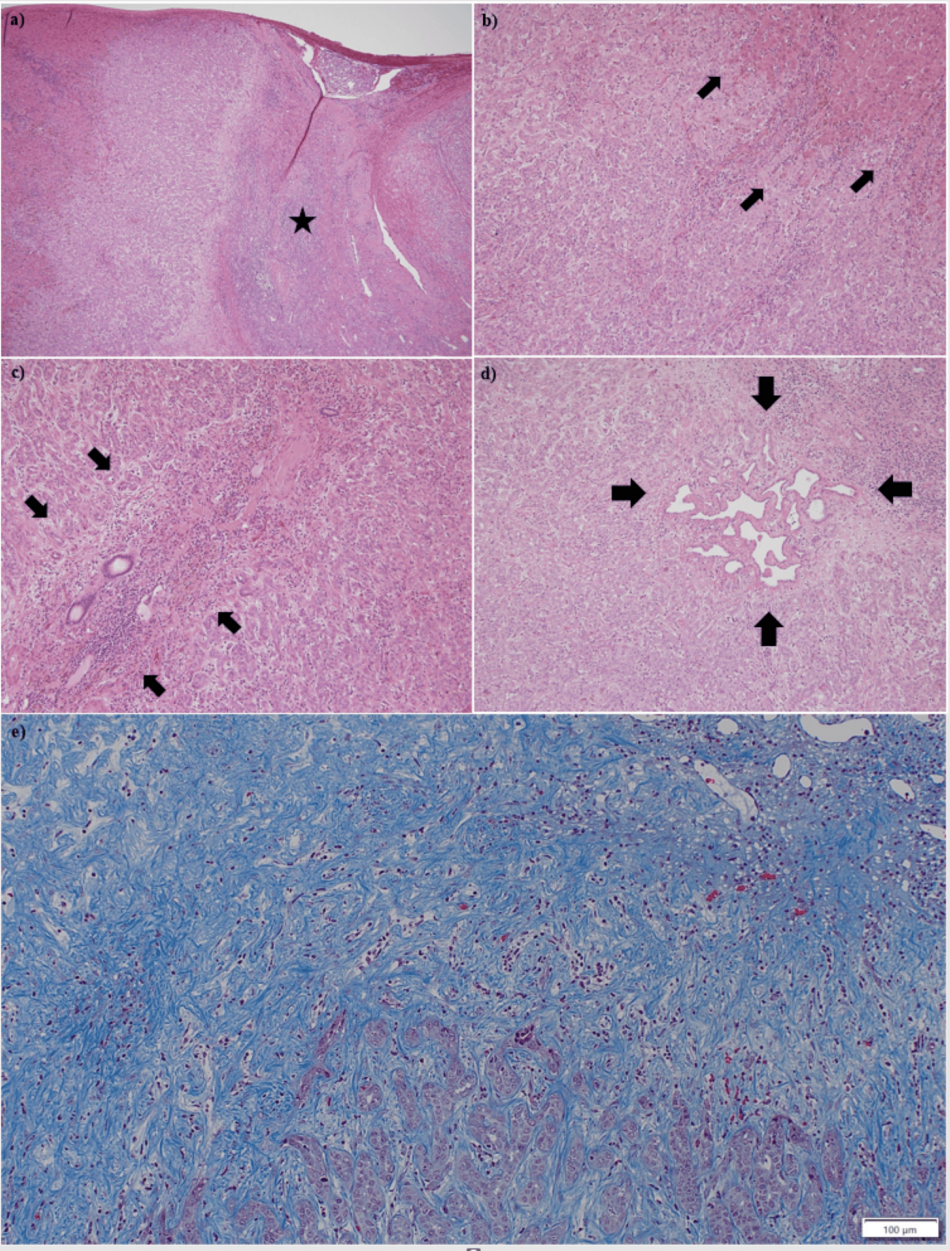
Microscopically findings of the tumor. Microscopically findings of the tumor. (a) The scar-like fibrosis in the central region of the tumor (asterisk) (hematoxylin-eosin stain, ×4). (b) Tumor lesions and non-neoplastic liver parenchyma exhibited interdigitating patterns (arrows) (hematoxylin-eosin stain, ×10). (c) A Glissonian sheath (arrows) existed in the scar-like fibrosis lesion (hematoxylin-eosin stain, ×10). (d) von Meyenbrug complex in the tumor (arrows) (hematoxylin-eosin stain, ×10). (e) A higher magnification image (Masson's trichrome stain, ×10) shows scar-like fibrosis in the central region of the tumor.

A partial hepatectomy of segment 8 was performed for the liver tumor. The 1.7 cm × 1.5 cm × 1.1 cm tumor was well-demarcated and located beneath the hepatic capsule (Figure 4). The tumor-cut surface was white and solid, with some areas exhibiting a pale yellow hue. Microscopically, findings showed the tumor lesion with scar-like fibrosis in the center (Figure 5a, 5e). The tumor had well-defined margins and lacked a capsule, and the tumor lesion and non-neoplastic liver parenchyma exhibited an interdigitating pattern in some areas (Figure 5a-5b). A Glissonian sheath existed at the scar-like fibrosis lesion (Figure 5c). About 1 mm von Meyenbrug complex existed in the tumor (Figure 5d).

**Figure 6:**
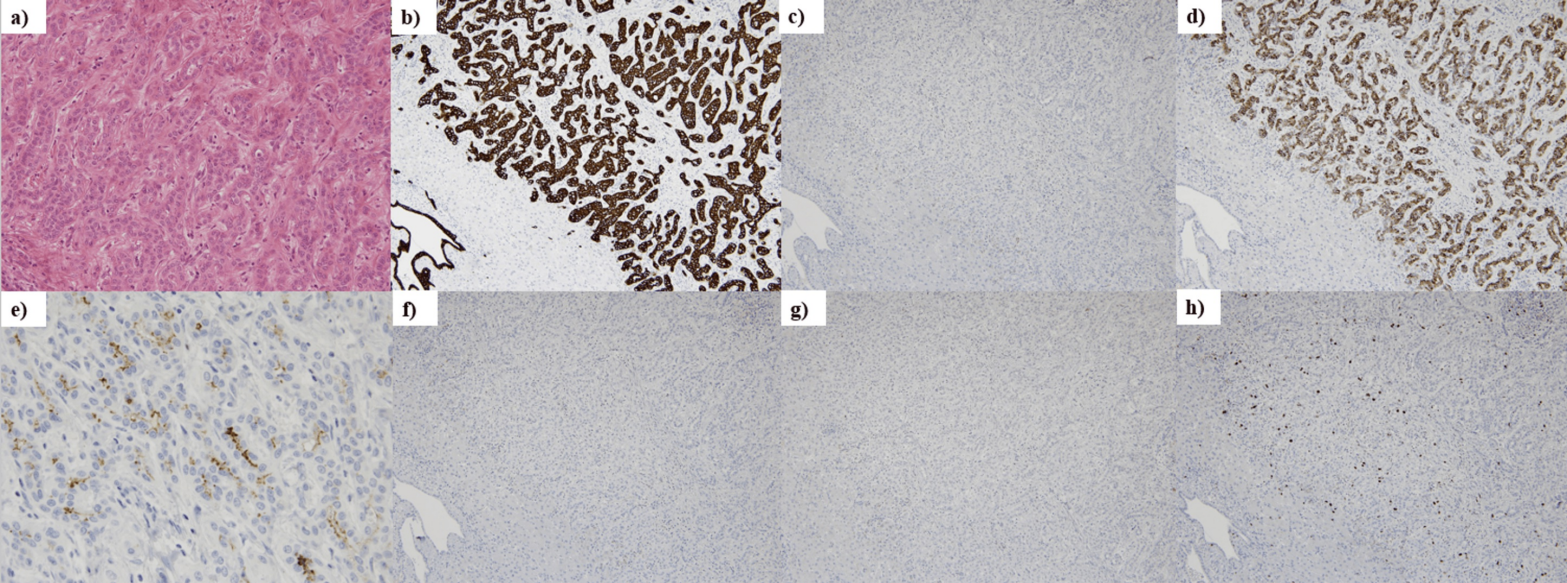
Immunohistochemical findings of the tumor. (a) The tumor exhibited dense proliferation of small glandular structures, each composed of a cluster of a few to several cells (hematoxylin-eosin stain, ×20). (b) Tumor cells were CK7 positive (×10), (c) CK20 negative (×10), (d) MUC6 positive (×10), (e) partially positive for EMA (×40), (f) negative for CD56 (×10), and (g) negative for p53 (×10). (h) The Ki-67 positivity rate was mostly less than 2%, with a maximum of 5% (×10).

The tumor exhibited dense proliferation of small glandular structures, each composed of a cluster of a few to several cells (Figure 6a). Immunohistochemical findings demonstrated positivity for both cytokeratin 7 (CK7) (Figure 6b) and mucin-6 (MUC6) (Figure 6d). Epithelial membrane antigen (EMA) staining revealed partial positivity on the endoluminal surface of tumor cells (Figure 6e). Ki-67 expression was assessed using immunohistochemistry, revealing a positivity rate of <2% with a maximum of 5% (Figure 6h). In contrast, staining for cytokeratin 20 (CK20), cluster of differentiation 56 (CD56), and p53 showed negative results in tumor cells (Figure 6c, 6f, 6g).

We diagnosed this hepatic tumor as a BDA based on the presence of Glissonian sheath within the tumor, positive CK7 and negative p53 immunohistochemistry, and a lower proliferative rate inferred from Ki-67 expression in the tumor cells. The patient remains alive with no recurrence of the tumor five years after hepatectomy.

## Discussion

Incidental findings, defined as new lesions detected on imaging procedures without prior related complaints, are frequently recorded [12-14]. This presents an opportunity for the early detection of tumor lesions, including malignant tumors. BDA, a rare benign hepatic tumor, is also one of the tumors incidentally detected by abdominal imaging. BDA, due to its rarity, is not mentioned in the latest guidelines for focal liver lesions [15]. Our presented BDA case was also incidentally discovered when the patient was examined for his chest computed tomography. Though focal hepatic lesions, including BDA, are necessary for differential diagnosis, there is no clear pathway for workup [14]. Especially for the healthy populations without significant medical background, a differential diagnosis is needed to use many examination modalities. According to epidemiological data on BDA, only one case was reported among Asians, compared to 108 cases among Caucasians and 10 cases among African Americans [1]. Due to the rarity of BDA in Asians, we were unable to diagnose BDA prior to surgical resection. Although no etiological factor for BDA has been identified, a reactive process to focal injury, such as postinflammatory or traumatic damage, has been hypothesized [1]. If inherent genetic anomalies caused BDA, we would likely see cases in children. However, the lack of such cases suggests that genetics may not be the primary cause.

BDA is an extremely rare benign tumor with no definitive imaging findings. Differential diagnosis between BDA and malignant tumors, including hepatocellular carcinoma and cholangiocarcinoma, is essential. Additionally, differentiation from other tumors like epithelioid hemangioendothelioma or hemangioma may be necessary. Its imaging may mimic hepatocellular carcinoma, showing enhancement in the arterial phase followed by a drop in the portal venous and delayed phases [2,8]. On the other hand, persistent enhancement on portal venous and delayed phases was shown in some BDA cases following imaging of enhancement in the arterial phase [5,6]. BDA appeared hypointense on T1-weighted images, hyperintense on T2-weighted images, and hyperintense on diffusion-weighted images [5-7,16]. However, some images [5,17] also show a hypointense signal on T2. Our case showed the aforementioned imaging findings, but we could not diagnose BDA based on these findings alone. Consistent with this, diagnosing BDA solely on imaging findings is difficult, and pathological confirmation is necessary for a precise diagnosis.

Histologically, BDA appeared as a well-circumscribed, non-encapsulated nodule [1]. The mass itself is composed of well-differentiated proliferating bile ducts; additionally, intra-tumoral fibrous tissue increases during tumor progression [2]. These characteristics were observed in our case. Immunohistochemical findings of BDA show CK7 positivity, indicating the involvement of bile duct epithelial cells [2,7]. Although p53 negativity is consistent with findings in previously reported benign tissues [2,7], it does not conclusively establish a benign diagnosis. This is because negative p53 staining does not necessarily imply the absence of a p53 mutation.

Ki-67 expression, a proliferation marker of tumors, exhibited a lower positivity rate in our cases. Given that Ki-67 protein expression is generally considered an absolute requirement for cell proliferation [18], its staining positivity is often associated with the malignant nature of a tumor. Moreover, higher Ki-67 expression in tumors is frequently correlated with elevated levels of other malignant biomarkers [19,20]. Consequently, the lower Ki-67 expression observed in our BDA cases strongly suggests that BDA is a benign disease.

Additionally, EMA staining is positive [1,2]. Our case was consistent with these characteristics, too. On the other hand, CD56, which shows positive for some kinds of carcinoma cells, was reported positive with BDA [7], though our case showed negative. These variations in CD56 expression may indicate that the disease concept of bile duct adenoma encompasses a wide range of pathological conditions, extending from completely benign to borderline neoplastic lesions.

Cholangiocarcinoma is one of the malignant diseases that must be considered in the differential diagnosis of BDA. Immunohistochemical typing may help differentiate BDA from cholangiocarcinoma. The immunohistochemical typing of CK19, CK20, MUC2, MUC5AC, CA19-9, CEA, CA125, and SMAD4 for the differentiation is suggested [10]. The measurement of serum α-fetoprotein and PIVKA-II may be useful for the differential diagnosis of hepatocellular carcinoma. Due to the non-specificity of BDA in terms of clinical features and imaging findings, these modalities are not useful for differentiating BDA from other malignant neoplasms, especially early cholangiocarcinoma. The clinical utility of genetic alteration detection for diagnosing BDA remains unknown.

For patients with BDA, a targeted biopsy of liver tumors may be a useful procedure due to its lower invasiveness compared to surgical resection [11,17]. However, some BDA cases with coexisting cholangiocarcinoma were reported [3,21]. And the co-existence of BDA and cholangiocarcinoma suggests a possible adenoma-carcinoma sequence [3,9]. Though genetic analysis studies have not conclusively proven that bile duct adenoma is a precursor to cholangiocarcinoma [22], some BDA are known to exhibit neoplastic potential followed by cholangiocarcinoma [23]. A high prevalence of BRAF V600E mutations was found in 53% of BDA, which may progress to cholangiocarcinoma [24]. BDA may be a diverse group of bile duct epithelial cells, including both normal and dysplastic cells. While pathological findings from targeted biopsy can be helpful for BDA diagnosis, sampling error may lead to misdiagnosis of BDA as a simple benign disease, especially when malignant tumor tissue is present within a portion of the BDA mass. Therefore, we recommend either surgical resection or strict follow-up with imaging for the BDA and its suspected lesion. However, specific long-term management may not be necessary for BDA in the absence of malignant tissues, as the prognosis for BDA is generally favorable. Nevertheless, BDA can develop in the context of hepatic steatosis [5], and in such cases, long-term follow-up is recommended to monitor for the development of any other hepatic malignant tumors, such as hepatocellular carcinoma.

## Conclusions

In conclusion, we encountered a single case of BDA. The diagnosis of BDA was made based on pathological findings following surgical resection of the liver tumor. While BDA is a rare and benign liver tumor, it carries a potential risk of harboring or developing malignant tissue, such as cholangiocarcinoma, in the future. Therefore, surgical resection is the recommended treatment for BDA. If surgery is not feasible, strict follow-up is crucial. If possible, the resected tumor should be examined for genetic mutations. If mutations associated with malignancy are detected, more intensive follow-up is necessary.
